# Supplementary material for: Short and Concise Peer-to-Peer Teaching—Example of a Successful Antibiotic Stewardship Intervention to Increase Iv to Po Conversion
Source: Antibiotics (Basel). 2022 Mar 17;11(3):402. doi: 10.3390/antibiotics11030402 (PMC8944614; doi:10.3390/antibiotics11030402)
Supplement: Supplementary file 1 [file antibiotics-11-00402-s001.zip › antibiotics-1638845-supplementary.pdf]

# Antibiotic therapies on the ward

**Not everyone needs i.v. therapy!**

# Uncomplicated urinary tract infection

- Oral whenever possible !
- as short term as possible !
- Quinolones and cephalosporins: OUT!

| Substance | Dose | Duration |
|---|---|---|
| Fosfomycin-Trometamol | 300mg 1 per day | 1 Day |
| Nitrofurantoin | 50mg 4 per day | 7 Days |
| Nitrofurantoin RT | 100mg 2 per day | 5 Days |

In patients with diabetes mellitus without other relevant diseases/complicating factors, urinary tract infections can be considered uncomplicated if the metabolic situation is stable

# Pyelonephritis

- Mild and moderately severe courses of pyelonephritis should be treated with oral antibiotics
  - antibiotic therapy for 5 to 10 days
  - Oral: cefpodoxime, ciprofloxacin, levofloxacin

- severe infections with accompanying systemic symptoms, such as nausea, vomiting, or circulatory instability
  - parenteral: ceftriaxone, ciprofloxacin, levofloxacin

# Pneumonia – Community acquired

Mild severity
- CRB-65: 0
- oxygenation not altered spO2 > 89%
- no decompensated comorbidity

Moderate severity
- between mild and severe

Severe pneumonia
- severe sepsis / septic shock
- respiratory insufficiency
- decompensated comorbidity

# Pneumonia – Community acquired

- Microbiological diagnostics:
  - at least 2 BK pairs
  - Urine AG test for Legionella
  - adequate sputum or TS/BS/BAL

- seasonal PCR for influenza (!)
- no (!) multiplex PCR for viral/bacterial pathogens

# Pneumonia – Community acquired

| | Disease Severity | Therapy | | |
|---|---|---|---|---|
| | | 1st Choice | | 2nd Choice |
| p.o. | **Mild Pneumonia without comorbidity** | Amoxicillin | | Moxifloxacin, Levofloxacin |
| | **oral therapy** | | | Clarithormycin, Azithromycin |
| | | | | Doxycycline |
| | **Mild Pneumonia with comorbidity** | Amoxicillin/Clavulanic Acid | | Moxifloxacin, Levofloxacin |
| | **oral therapy** | | | |
| | chronic heart disease | | | |
| | CNS disease with dysphagy | | | |
| | COPD | | | |
| | reduced mobility | | | |
| i.v. – p.o. | **Moderate Pneumonia** | Amoxicillin/Clavulanic Acid | w/wo Makrolid for 3 days | Moxifloacin, Levofloxacin |
| | **start with iv - sequential oral therapy** | Ampicillin/Sulbactam | w/wo Makrolid for 3 days | |
| | | Cefuroxime | w/wo Makrolid for 3 days | |
| | | Ceftriaxone | w/wo Makrolid for 3 days | |
| | | Cefotaxime | w/wo Makrolid for 3 days | |
| i.v. | **Severe Pneumonia** | Piperacillin/Tazobactam | with Makrolid for 3 days | Moxifloxacin, Levofloxacin (no montherapy in septic shock) |
| | **start with iv - sequential oral therapy if possible** | Ceftriaxone | with Makrolid for 3 days | |
| | | Cefotaxime | with Makrolid for 3 days | |

# Pneumonia – Community acquired

| Disease Severity | Therapy | | |
|---|---|---|---|
| | 1st Choice | | 2nd Choice |
| | | | |
| **Mild Pneumonia without comorbidity** | Amoxicillin | | Moxifloxacin, Levofloxacin |
| **oral therapy** | | | Clarithormycin, Azithromycin |
| | | | Doxycycline |
| | | | |
| **Mild Pneumonia with comorbidity** | Amoxicillin/Clavulanic Acid | | Moxifloxacin, Levofloxacin |
| **oral therapy** | | | |
| chronic heart disease | | | |
| CNS disease with dysphagy | | | |
| COPD | | | |
| reduced mobility | | | |
| | | | |
| **Moderate Pneumonia** | Amoxicillin/Clavulanic Acid | w/wo Makrolid for 3 days | Moxifloacin, Levofloxacin |
| **start with iv - sequential oral therapy** | Ampicillin/Sulbactam | w/wo Makrolid for 3 days | |
| | Cefuroxime | w/wo Makrolid for 3 days | |
| | Ceftriaxone | w/wo Makrolid for 3 days | |
| | Cefotaxime | w/wo Makrolid for 3 days | |
| | | | |
| **Severe Pneumonia** | Piperacillin/Tazobactam | with Makrolid for 3 days | Moxifloxacin, Levofloxacin (no montherapy in septic shock) |
| **start with iv - sequential oral therapy if possible** | Ceftriaxone | with Makrolid for 3 days | |
| | Cefotaxime | with Makrolid for 3 days | |

**Pathogen spectrum basically the same for all severity levels**

# Pneumonia – Community acquired

- Mild pneumonia with defined comorbidity
- Combination penicillin with BLI:
  - Spectrum expansion against ß-lactamase-producing S. aureus, H. influenzae, and enterobacteria
  - Caution Unacid: dose of penicillin component too low
- Clarithromycin?
  - Initial combination for three days
  - then discontinue after negative rapid Legionella test
  - **P.o. bioavailability extremely good (also Clinda and Levo)!**

# Pneumonia – Community acquired

Duration :

mild to moderate CAP: 5-7 days, shorter may be possible.

before end of therapy always clinical stabilization for at least 2 days

oral sequential therapy or directly oral!

Severe CAP: up to 7 days

before end of therapy always clinical stabilization for at least 2 days

oral sequential therapy if necessary

initially at least 3 days parenterally

# Oral sequential therapy: criteria

- Heart rate < 100 / min
- Respiratory rate < 25 / min
- Systolic blood pressure > 90 mmHg
- Body temperature < 37.8 oC
- Ability to take in food orally
- Normal state of consciousness
- No hypoxemia (SpO2 > 90%, PaO2> 60 mmHg)
